# Effect of particulate matter 2.5 on QT dispersion in patients with chronic respiratory disease

**DOI:** 10.1038/s41598-022-18355-w

**Published:** 2022-08-18

**Authors:** Wanwarang Wongcharoen, Thanaphum Uthaithummakul, Sarunsorn Krintratun, Thananan Thongsujaritkul, Thanatat Wattananukorn, Teerapat Nantsupawat, Chaicharn Pothirat, Juthamas Inchai, Arintaya Phrommintikul

**Affiliations:** 1 Department of Internal Medicine, Faculty of Medicine, Chiang Mai University, Chiang Mai, Thailand; 2 Center for Medical Excellence, Faculty of Medicine, Chiang Mai University, Chiang Mai, Thailand

**Keywords:** Cardiology, Health care

## Abstract

PM2.5 air pollutants increased risk of ventricular arrhythmias. The prolonged corrected QT interval (QTc) and QT dispersion (QTd) is common in patients with chronic airway disease and is associated with heightened risk of ventricular tachyarrhythmia. We sought to examine the effect of PM2.5 exposure on QTc and QTd in patients with chronic airway disease. We enrolled 73 patients with chronic airway disease into the study. The 12-lead ECGs were recorded during high-exposure and low-exposure periods of PM2.5. QTc and QTd were compared between 2 periods. Mean age was 70 ± 10 years. Mean FEV1/FVC was 63 ± 14%. There was no difference in QTc between PM2.5 high-exposure and low-exposure periods. However, QTd was significantly increased during PM2.5 high-exposure compared to low-exposure periods in male patients (43.5 ± 15.0 vs. 38.2 ± 12.1 ms, P = 0.044) but no difference was found in females. We found that patients who worked mostly indoor had less QTd than those working outdoor during PM2.5 low-exposure period. In addition, those who wore face mask tended to have less QTd during low-exposure period than those who did not. High PM2.5 exposure increased QTd in male patients with chronic airway disease. Working indoors and wearing face mask were associated with less QTd.

## Introduction

Air pollution has become a great concern for environmental health issues worldwide. Several studies have demonstrated that air pollution has negative health effects in patients with various conditions. Air pollution is a complex mixture of gaseous and particulate materials. It has been shown that particulate matter (PM) air pollutants increased susceptibility to airway diseases including chronic obstructive pulmonary disease (COPD) and asthma^1,2^. In addition, PM air pollutants have been shown to increase the risk of various cardiovascular diseases^3,4^. Previous studies have demonstrated that PM with an aerodynamic diameter of ≤ 2.5 µM (PM2.5) was associated with a more robust association with cardiac arrhythmias compared to other air pollutants^5^.

Cardiovascular diseases are frequent co-morbidities found in patients with chronic airway disease and account for 30–50% of mortality in this population^6^. On the other hand, chronic airway diseases have also been shown to increase risk of cardiovascular diseases^7^. It is well-known that prolongation of corrected QT interval (QTc) is associated with an increased risk of ventricular arrhythmias and sudden cardiac death. The QT dispersion (QTd) indicates heterogeneity of ventricular repolarization and has also been shown to predict long-term risk of arrhythmias and cardiovascular mortality^8^. Previous study has demonstrated that prolongation of QTc and QTd is not uncommon in patients with COPD^9^. Of note, the increased QTd has been shown to enhance the risk of ventricular arrhythmias in patients with COPD^10^.

Several investigators have explored the effect of air pollution on QTc interval^11–13^. They found that PM air pollutants were associated with increased QTc interval in healthy adults^12^ and elderly population^11^. Nevertheless, the effect of PM2.5 exposure on QTc has never been studied in patients with chronic airway disease. Also, the effect of PM2.5 air pollutants on QTd has never been explored in any population. Therefore, we sought to examine the effects of PM2.5 exposure on QTc and QTd in patients with chronic airway disease.

## Method

A prospective observational study was conducted between March and August 2016 in Chiang Dao district. We enrolled 73 patients with chronic airway disease visiting the outpatient clinic in Chiang Dao hospital. Chiang Dao is one of the districts in Chiang Mai, Thailand, facing a high level of seasonal air pollution every year. The PM concentration in Chiang Mai, Thailand, has revealed a similar pattern every year since 2010. The high level of PM concentration starts at the beginning of December, reaches its peak in March, and subsequently decreases from May to November. The sampling station was located in Chiang Dao hospital, Chiang Mai, Thailand. The ambient air concentration of pollutants was measured by the DustDETECT™. The analytical method for PM was specifically designed to monitor the flow of particulate emissions from small stacks and emission points being passed through an air filtration system. Table 1 shows the pollutants data between the PM2.5 high-exposure period (March 2016) and the low-exposure period (August 2016).

**Table 1:** Pollutants data between high-exposure period and low-exposure period.

Pollutants data	High-exposure period	Low-exposure period
PM2.5 (µg/m^3^)	74 (31–138)	14 (8–23)
PM10 (µg/m^3^)	108 (53–209)	30 (19–48)
SO_2_ (µg/m^3^)	1 (1–3)	1 (1–2)
NO_2_ (µg/m^3^)	32 (5–88)	13 (2–32)
CO (µg/m^3^)	1.33 (0.5–4.4)	0.84 (0.4–2.4)

*CO* carbon monoxide, *NO*_*2*_ nitrogen dioxide, *PM* particulate matter, *SO*_*2*_ sulfer dioxide.

In this study, 12-lead electrocardiograms (ECGs) (HP M1700A, Hewlett Packard, Palo Alto, CA, USA) of patients were recorded during the high- and the low-exposure period of PM2.5. The ECGs were recorded at a paper speed of 25 mm/s. The parameters measured including heart rate, QT interval, QTc interval, and QTd. The QT interval was measured from the earliest QRS deflection to the end of the T wave in all leads. The QT interval was corrected for heart rate using Bazett’s formula (QTc = QT/√RR). QTd was defined as the maximum QT interval minus the minimum QT interval measured on any leads of the ECGs. All ECGs were analyzed for QT parameters by blinded investigators using a digital caliper. Previous study showed that the mean number of ECG leads that QT interval could be analyzed was 9.9 ± 1.5 leads^14^. As a result, a minimum of 9 ECG leads were required to analyze QT interval in the present study. The QTc and QTd were compared between those measured during the PM2.5 high-exposure period (March 2016) and the low-exposure period (August 2016).

### Statistical analysis

Continuous variables were expressed as means and standard deviations when normally distributed, or medians and interquartile ranges when not normally distributed. Categorical variables were presented as frequency (%). The numerical variables were compared within groups with paired t test or Wilcoxon matched paired sign-rank test. Statistical software package IBM SPSS Statistics for Windows, version 23.0 (IBM Corp., Armonk, NY, USA, https://www.ibm.com/products/spss-statistics) was used for analysis.

### Ethics approval and consent to participate

The effect of PM2.5 on QT dispersion in patients with chronic airway disease was approved by the ethics committee of the Faculty of Medicine, Chiang Mai University, approval number 536/2558 The investigations were carried out in accordance with the Declaration of Helsinki, including written informed consent of all participants.

## Results

A total of 73 patients were included in the study. Mean age was 70 ± 10.0 years (range from 42 to 87 years), and 39 (53.4%) patients were male. Body mass index was 20.9 ± 4.3 kg/m^2^ (range from 14.1 to 32.9 kg/m^2^). Of the 73 patients, 52 (71.2%) had COPD, 13 (17.8%) had asthma and 8 (11.0%) had other airway diseases. The mean forced vital capacity (FVC) was 2.2 L ± 0.7%, and the mean forced expiratory volume in one second (FEV1)/FVC was 63.4 ± 13.8%. The prevalence of hypertension, diabetes mellitus, and dyslipidemia was 53.4%, 6.8%, and 21.9%, respectively. Table 2 shows baseline characteristics of the studied population.

**Table 2:** Baseline characteristics of the studied population.

Characteristics	N = 73
Age (years)	70 ± 10.0
Male, n (%)	39 (53.4)
BMI (kg/m^2^)	20.9 ± 4.3
**Pulmonary function test**	
FVC (l)	2.2 ± 0.7
Percent predicted of FVC (%)	92.5 ± 26.2
FEV1 (l)	1.4 ± 0.7
Percent predicted of FEV1 (%)	74.3 ± 29.1
FEV1/FVC (%)	63.3 ± 13.8
Current or ex-smoker, n (%)	62 (84.9)
**Comorbidities**	
Respiratory disease	
COPD, n (%)	52 (71.2)
Asthma, n (%)	13 (17.8)
Other airway diseases, n (%)	8 (11)
Hypertension, n (%)	39 (53.4)
Diabetes mellitus, n (%)	5 (6.8)
Dyslipidemia, n (%)	16 (21.9)
Stroke, n (%)	4 (5.5)
Chronic kidney disease, n (%)	3 (4.1)

*BMI* body mass index, *COPD* chronic obstructive pulmonary disease, *FEV1* forced expiratory volume in 1 s, *FVC* forced vital capacity.

There was no difference in QTc between the PM2.5 high-exposure and the low-exposure periods in both male (426.8 ± 30.9 ms vs. 433.4 ± 37.0 ms, P = 0.422) and female patients (442.5 ± 20.9 vs. 436.9 ± 25.3 ms, P = 0.193). Interestingly, QTd was significantly greater during the PM2.5 high-exposure period than that measured during the low-exposure period in male patients (43.5 ± 15.0 vs. 38.2 ± 12.1 ms, P = 0.044). While there was no difference in QTd between the 2 periods in female patients (39.6 ± 14.0 vs. 38.0 ± 13.0 ms, P = 0.565) (Table 3).

**Table 3:** Differences in corrected QT interval and QT dispersion during the PM2.5 high-exposure and the low-exposure period.

QT parameters	High-exposure period	Low-exposure period	P value
**Male (N = 39)**			
QTc interval (ms)	426.8 ± 30.9	433.4 ± 37.0	P = 0.422
QT dispersion (ms)	43.5 ± 15.0	38.2 ± 12.1	P = 0.044
**Female (N = 34)**			
QTc interval (ms)	442.5 ± 20.9	436.9 ± 25.3	P = 0.193
QT dispersion (ms)	39.6 ± 14.0	38.0 ± 13.0	P = 0.565

*QTc* corrected QT interval.

There were 33 (45.2%) patients who reported working mostly indoors. In addition, there were 38 (52.0%) patients reported wearing face mask most of the time. We found that patients who worked mostly indoors had less QTd than those working outdoors during the PM2.5 low-exposure period (34.7 ± 15.0 vs. 41.0 ± 10.1, P = 0.042). However, during the PM2.5 high-exposure period, QTd did not differ between those who worked indoors and outdoors (40.3 ± 16.6 vs. 42.9 ± 12.8 ms, P = 0.452). Furthermore, those who wore face mask tended to have less QTd during the PM2.5 low-exposure period than those who did not wear face mask (35.9 ± 12.6 vs. 40.6 ± 12.9 ms, P = 0.072). Nevertheless, during the PM2.5 high-exposure period, QTd was not different between those who wore the mask and those who did not (43.3 ± 14.4 vs. 40.0 ± 14.8 ms, P = 0.343).

Of note, we demonstrated that male patients were more likely to work outdoors than female patients (64.1% vs. 44.1%, P = 0.087). However, male and female patients wore face mask in a similar fashion (56.4% vs. 47.4%, P = 0.425).

## Discussion

It has been widely recognized that ambient air pollution contributes to adverse health impacts. Exposure to PM2.5 accounts for the enhanced risk of various health conditions, including cardiovascular diseases^4,5^. Several investigators have demonstrated the link between PM2.5 and increased risk of cardiac arrhythmias, including bradycardia, premature contractions, atrial tachyarrhythmias, and ventricular arrhythmias^5,15,16^.

PM pollution is a major detrimental risk factor for COPD and other chronic airway diseases^17^. Previous studies have demonstrated that PM2.5 is associated with decreased lung function and increased risk of hospitalization and mortality in patients with COPD^18,19^. Cardiovascular disease is a significant contributor to morbidity and mortality in patients with COPD and other airway diseases^6^. The increase in QTc and QTd interval has been described in patients with COPD. One-third of patients with COPD had prolonged QTc interval and a quarter of them had increased QTd compared to healthy subjects^9^. The increased QTd observed in patients with COPD was found to heighten the risk of ventricular arrhythmias^10^.

In the present study, we found that there was no difference in QTc interval between the high-exposure and the low-exposure periods of PM2.5 in patients with chronic airway disease. Our results were in contrast to other studies which demonstrated that PM2.5 increased QTc interval in healthy subjects and elderly population^11–13^. The disparity of the results may be attributable to the dissimilarity of the studied population. It has been shown that patients with COPD had an increased QTc interval compared to healthy subjects. It is plausible that PM2.5 may have less effect on QTc interval in patients with COPD compared to others. Furthermore, previous study has shown that one-year PM2.5 exposure significantly increased QTc interval and demonstrated a strong positive correlation between QTc prolongation and the longer PM2.5 exposure time^11^. Our studied population has exposed to a high level of PM2.5 for approximately 4 months each year. The difference in temporal and geographical distribution of PM2.5 exposure among the studies may have partly explained the different results.

Interestingly, we demonstrated an increase in QTd during the high-exposure period of PM2.5 compared to the low-exposure period. QTd reflects heterogeneity of ventricular repolarization and is associated with the enhanced risk of ventricular arrhythmia and sudden cardiac death^8^. However, this finding was observed only in male patients. There was no difference in QTd in females between 2 periods. It is unclear whether PM2.5 exposure has a different effect on gender. We hypothesized that male patients might have exposed to a greater magnitude of PM2.5 due to the fact that males in our studied population were more likely to work outdoors as compared to females.

Furthermore, we demonstrated that patients who worked indoors had less QTd during the PM2.5 low-exposure period but no difference was found during the high-exposure period. The reason is unknown. We speculated that working indoors may have a slow protective effect on the hazard of PM2.5 exposure by shortening of QTd after PM2.5 exposure. We also observed a similar effect of QTd shortening during the PM2.5 low-exposure period in patients who wore mask compared to those who did not.

The mechanisms underlying PM2.5 effect on the alteration of ventricular repolarization have been proposed. Due to the very small-sized particles, it has been shown that PM2.5 can enter systemic circulation^20^. Previous animal study suggested that the prolongation of action potential duration resulted from PM air pollution may be mediated by oxidative stress and calcium calmodulin kinase II activation^21^. Furthermore, several investigators have shown that PM2.5 exposure was associated with the change of autonomic nervous system leading to sympathovagal imbalance^22,23^. The PM2.5 effect on vasomotor disturbance, hypercoagulability, and adverse metabolic effect has been described^3,24,25^. This may result in myocardial ischemia leading to the heterogeneity of ventricular repolarization.

There were several limitations in our study. First, the sample size was relatively small to draw the definitive conclusion from the present study. Larger studies are warranted to confirm our results. Second, we did not have data on climatic conditions during PM2.5 high-exposure period and low-exposure period. Whether the different climatic conditions may have an effect on the QTc and QTd intervals is unknown and needs further study. Third, we did not have detailed data on medications used in our studied population. The medications used may have changed during 5-month period in some patients which may have affected the QTc and QTd intervals. With this regard, the results of the present study can be subject to bias from unmeasured confounding and should be interpreted with caution.

## Conclusion

We demonstrated that high exposure to PM2.5 increased QTd in male patients with chronic airway disease. This detrimental effect of PM2.5 may lead to an increased risk of ventricular arrhythmias and mortality. In addition, working indoors and wearing face mask were associated with less QTd during the low-exposure period. These protective strategies should be encouraged in patients with chronic airway disease exposing to a high level of PM2.5.

## Data Availability

The informed consent given by Effect of PM2.5 on QT dispersion in patients with chronic airway disease study participants does not cover data posting in public databases. However, data are available upon request should be sent to arintaya.p@cmu.ac.th and are subject to approval by the Faculty of Medicine, Chiang Mai University Ethics Committee.
